# The mutation of Japanese encephalitis virus envelope protein residue 389 attenuates viral neuroinvasiveness

**DOI:** 10.1186/s12985-024-02398-8

**Published:** 2024-06-05

**Authors:** Rong Huang, Yajing He, Chenghua Zhang, Yue Luo, Chen Chen, Ning Tan, Yang Ren, Kui Xu, Lei Yuan, Jian Yang

**Affiliations:** 1 Institute of Basic Medicine and Forensic Medicine, North Sichuan Medical College, Nanchong, 637100 China; 2 School of Pharmacy, North Sichuan Medical College, Nanchong, 637100 China

**Keywords:** Japanese encephalitis virus, Neuroinvasiveness, E protein, E389, Molecular conformation

## Abstract

The envelope (E) protein of the Japanese encephalitis virus (JEV) is a key protein for virus infection and adsorption of host cells, which determines the virulence of the virus and regulates the intensity of inflammatory response. The mutation of multiple aa residues in the E protein plays a critical role in the attenuated strain of JEV. This study demonstrated that the Asp to Gly, Ser, and His mutation of the E389 site, respectively, the replication ability of the viruses in cells was significantly reduced, and the viral neuroinvasiveness was attenuated to different degrees. Among them, the mutation at E389 site enhanced the E protein flexibility contributed to the attenuation of neuroinvasiveness. In contrast, less flexibility of E protein enhanced the neuroinvasiveness of the strain. Our results indicate that the mechanism of attenuation of E389 aa mutation attenuates neuroinvasiveness is related to increased flexibility of the E protein. In addition, the increased flexibility of E protein enhanced the viral sensitivity to heparin inhibition in vitro, which may lead to a decrease in the viral load entering brain. These results suggest that E389 residue is a potential site affecting JEV virulence, and the flexibility of the E protein of aa at this site plays an important role in the determination of neuroinvasiveness.

**Supplementary Information:**

The online version contains supplementary material available at 10.1186/s12985-024-02398-8.

## Introduction

Japanese encephalitis (JE) is a serious neurological disease caused by the Japanese encephalitis virus (JEV) that has become a major health concern in Asian countries.Children under the age of 15 years are particularly vulnerable to JE, with a mortality rate of up to 30% [1], and nearly half of survivors develop severe neurological and mental sequelae later in life [2].

JEV is a plus-sense, single-stranded RNA virus belongs to the Flavivirus genus, which includes Dengue virus (DENV), Zika virus (ZIKV), West Nile virus(WNV), and Murray Valley Encephalitis virus (MVEV). The JEV genome is approximately 11 kb in length and contains untranslated regions at the 5’ and 3’ ends as well as an open reading frame encoding a single polyprotein, which is cleaved into three structural proteins (C, prM/M and E) and seven nonstructural proteins (NS1, NS2A, NS2B, NS3, NS4A, NS4B and NS5) by the combined action of viral and host proteases [3]. As the only mature glycosylated protein, E protein plays a crucial role in virus and receptor cell adsorption, facilitating membrane fusion and boosting virus entrance into cells, and is thought to have a large impact on the virulence of JEV. As a result, the E protein has traditionally been the primary focus of the JEV virulence research [4–6]. The genome sequences of numerous JEV strains with varying virulence are compared, and it is determined that individual aa alterations in the E protein affect JEV neurovirulence or neuroinvasiveness but lack some important virulence attenuation mechanisms. Studies have confirmed that the Glu-to-Lys mutation at the 138 aa position of the E protein in vaccine strain SA14-14-2 plays an important role in reducing its neurovirulence, and it is worth noting that the substitution of aa in this area also increases the attenuated strain’s binding affinity to glycosaminoglycans (GAGs) [7, 8].

During the viral infection, its protein structures undergo a series of conformational changes that affect the binding of the virus to the receptor or antibody recognition, further producing virulence differences. Using molecular dynamics modeling methods, it is possible to elucidate the underlying mechanisms that lead to alterations in molecular flexibility and receptor or antibody recognition. The mutations in E protein T198F of WNV can regulate the molecular conformational dynamics of the virus, exposing distal neutralizing epitopes and thus regulating the efficacy of viral antibody recognition [9].

The spatial structure of the E protein revealed that it was composed of three domains (DI, DII, and DIII), with DIII containing a large number of unique epitopes and epitopes implicated in cell receptor binding being mostly concentrated in this region [5]. JEV infects via receptor-mediated endocytosis. However, its specific receptor has yet to be discovered. According to some researchers, flaviviruses can exploit GAG, which is abundantly dispersed on the cell surface and in the extracellular matrix, to mediate viral attachment to and infection of target cells [8, 10, 11]. The adaptation of flavivirus to tissue culture increases the positive charge gain of E protein, resulting in increased affinity for GAG. A probable link between enhanced affinity for GAG and lower virulence was hypothesized in a study of MVE-attenuated strains [12]. We extended the study on the influence of alterations in viral GAG affinity on virulence attenuation to JEV because of the substantial similarity of JEV and MVE E protein structures.

In the previous experiment, by successive passage of WT strain JEV SA14 in BHK21 cells, we measured the LD_50_ (PFU) values of the harvested virus and WT (JEV SA14). The results showed that the harvested virus exhibited lower LD_50_ (10^3^) values of neuroinvasiveness as compared with that of the WT (JEV SA14) (LD_50_ = 2.1), whereas the harvested virus exhibited similar LD_50_ values of neurovirulence to that of JEV WT reported by others [13]. In order to find out the explanation for the reduced neuroinvasiveness, the full-length genome of the harvested virus was sequenced, and it was found that a mutation from A to G occurred at site 1166 of the E protein, which resulted in the mutation of aa 389 of the E protein from aspartate (Asp) to glycine (Gly). The results showed that changing the aa E389 might lessen the neuroinvasiveness of JEV. Here, we investigated the impact of three distinct aa residues (D389G, D389S, and D389H) at E389 on the neuroinvasiveness of JEV, as compared with the virulent WT (JEV SA14). Our results demonstrated that the flexibility of the E protein of the E389 aa residue plays an important role in the neuroinvasiveness of JEV.

## Materials and methods

### Cells and viruses

BHK-21 (baby hamster kidney), Vero (African green monkey kidney), and U87 (human glioma) cells were cultivated in Dulbecco’s modified Eagle’s medium(DMEM)supplemented with 10% fetal bovine serum in a humidified 5% CO_2_ atmosphere at 37 °C. The JEV SA14 strain (GenBank accession No. U14163) was created and kept in our lab before being passed into BHK-21 cells.

### Construction of infectious clone

All full-length cDNA clones of JEV mutants were constructed using a two-plasmid system, as described previously [13]. One plasmid pACNR-JEV (SA14)5’ contained a 3.4 kb fragment at the 5’ end of strain SA14, and the other contained a 7.5 kb fragment at the 3’ end of strain SA14[pACNR-JEV (SA14)3’].To acquire a fragment with an aa mutation at position 389 of the E protein, three sets of primers (Table S1) and PCR-based site-directed mutagenesis were used. KasI and BglII cut the fragment enzymatically to replace the matching location of the original plasmid pACNR-JEV (SA14)5’. It was possible to obtain the plasmid pACNR-JEV (SA14) 5’ (D389G, D389S, D389H). Subsequently, pACNR-JEV (SA14)3’ plasmid was digested with BspEI and XhoI enzymes, and a 7.5 kb JEV fragment from the digested product was cloned into pACNR-5’JEV to obtain full-length cDNA plasmid pACNR-JEV SA14 (D389G, D389S, D389H) containing the mutation site. All plasmids were sequenced to verify the engineered mutations.

### Rescue of mutant JEVs

The infectious clone plasmid was linearized by restriction digest using *XhoI* (NEB) and used as a template for in vitro transcription with Ribo m7G cap analog (Promega) using the Ribo MAX large-scale RNA production system Sp6 kit (Promega). The reaction system was incubated at 37 °C for 4 h. DNase I (Promega) was used to treat the products, followed by purification with the RNeasymini kit (Qiagen). BHK-21 cells (ATCC, CCL-10) were washed twice with PBS, and then 5 × 10^6^ cells (200 µl) were mixed with RNA (1 µg) synthesized in vitro under the condition of 270 V and pulse width 1500 us using an Electric transfer device (X-Porator H1, Etta Biotech, China) pulsed six times per 1000 ms. The transfected BHK-21 cells were cultivated in Dulbecco’s modified Eagle’s medium (DMEM, Boster, China) supplemented with 10% heat-inactivated fetal bovine serum (Tianhang Biology, China) at 37℃ in a 5% CO_2_ incubator. The virus was harvested when the cytopathic effect reached 30%. The harvested viruses were passaged two additional times in BHK-21 cells and titrated for the plaque assay. Three plaques with average diameter (about 2 mm) were selected and inoculated in BHK-21 cells. After 3 times of purification, the mutants were cultured in BHK-21 cells for subsequent experiments.

### Viral plaque assay and growth kinetics

The WT JEV SA14 and mutants were continuously diluted 10-fold in DMEM and the infected confluent monolayer of BHK-21 cells in a 6-well plate. After 1 h of absorption at 37 °C, viral inocula were removed, the agarose (low melting gel)(Solarbio, China) overlay in DMEM plus 2% FBS was added, and the cultures were maintained at 37 °C for 4 d. Plaques were stained with 0.5% violet after being fixed with 4% formalin. The virus titer is calculated by multiplying the number of plaques by the virus solution dilution.

BHK-21 and U87 cells were infected with the mutations or parental viruses at a multiplicity of infection (MOI) of 0.01 to evaluate the growth dynamics of WT and mutant viruses. After 1 h of absorption at 37℃, the inoculants were removed, and 20 ml DMEM supplemented with 2% inactivated fetal bovine serum was added. Every 12 h after infection, the culture supernatant was collected until cell death. Titers of the collected viruses were determined as described for the plaque assay.

### Indirect immunofluorescence assay (IFA)

The confluent monolayer of BHK-21 cells in 96-well plates was infected with the mutant viruses with an MOI of 0.01 and propagated for 48 h. WT strain JEV SA14 was tested as the WT virus. Cells were fixed with 4% formalin for 30 min and permeabilized with 0.1% Triton X-100 at room temperature for 10 min. Cells were washed with phosphate-buffered saline (PBS) and then incubated with primary antibodies (Abcam, monoclonal antibodies specific for JEV NS1 protein, 1:1000 dilution) at 37 °C for 1 h. After washing the cells with PBS three times, the secondary antibodies (Abcam, goat anti-mouse affinity-purified immunoglobulin G, 1:100 dilution) were added and incubated at 37℃ for 1 h. The fluorescent signals were visualized with a microscope and images were recorded using a SPOT camera (Olympus) after staining with 4’,6-diamidino-2-phenylindole (DAPI) for 5 min.

### Sequencing of JEVs

Continuous passage of mutants for ten rounds in BHK-21 cells to verify their stability. The viral RNA was extracted from the viruses using the RNA simple Total RNA Kit (TIANGEN, China), and the initial cDNA was synthesized using Prime Script™ RT Master Mix (Takara, China). To amplify the sequences at positions 1675–3004, including the E protein gene, the primers JEVCX2-F and JEVCX2-R, as well as high-fidelity polymerase (New England Biolabs), were utilized. PCR products were purified using the Gel Extraction Kit (OMEGA) and sequenced.

### RT-PCR detection

Total RNA was isolated from tissue or blood using the RNA simple Total RNA Kit (TIANGEN, China), and the initial cDNA was generated using the Prime Script™ RT Master Mix (Takara, China). The genomic copies were measured by using primers JEV NS5-F and JEV NS5-R. The Standard curves were made with plasmids incorporating 10-fold serial doses of the JEV E gene.

### Heparin-binding competition assay

To assess the inhibition effect of heparin on different mutants binding to BHK-21 cells, the mutants were pre-incubated at 37 °C for 30 min with low and high concentrations of heparin (0.1 and 1000 µg/ml) or mock-treated, cooled at 4℃ for 5 min.BHK-21 cells were cultured in 6-well plates, washed twice with pre-cooled PBS, and 2 ml DMEM was added to each well. The cells were placed at 4 °C for 30 min and then inoculated with the combination mentioned above, followed by incubation at 4 °C for a further 1 h. After washing three times with pre-cooled PBS, the RNA Simple total RNA kit (Tiangen, China) was used to isolate total RNA from viruses that bound to cells.

### Mouse experiments

To assess the neurovirulence of the JEV mutants, six mice per group of 3-week-old SPF Kunming mice were inoculated by the intracerebral (i.c.) route with 0.03 ml of 10-fold dilutions of the mutants or the WT virus, and inoculated mice were monitored for 14 d. The median lethal dose (LD_50_) was determined by the Reed and Muench calculation. Neuroinvasiveness was measured by inoculating 3-week-old SPF Kunming mice with 0.1 ml of 10-fold dilutions of the mutants or the WT virus by the intraperitoneal(i.p.) route, and the results were also recorded as LD_50_ (log_10_PFU). Mice in a moribund condition were euthanized and recorded as deaths.

To evaluate the mutant virus replication in mouse brains and peripheral tissues, 25 mice per group were inoculated i.p. with 10^6^ PFU mutants and WT viruses, respectively. Blood was collected daily from the orbital plexus on 1 to 5 dpi and at the times of liver and spleen tissue collection. At 6, 7, and 8 dpi, three mice in each group were randomly sacrificed by cervical dislocation, and the brains were removed. All tissues were snap-frozen immediately on liquid nitrogen immediately after extraction. The viral loads were detected by qRT-PCR.

The 10^6^PFU of mutants and WT virus were inoculated by intravenous (i.v.) route into 6-week-old female Kunming mice (*n* = 3) to assess virus clearance from the bloodstream. Blood was taken from the orbital plexus of animal each group at 5, 30 min, and 1 h p.i. after inoculation and allowed to clot at room temperature, and serum was collected by centrifugation at 4 °C, 3500/rpm for 15 min and stored at -80 °C. The viral loads at serum samples were determined by qRT-PCR.

### Molecular dynamics simulations

Using PDB ID 5WSN as a template [14], Swiss-Model [15] homology modeling was used to obtain the E protein structure of JEV (sequences share 98.80% similarity). By overlapping the E protein structure of JEV with the PDB ID 5WSN, a wild-type (WT) heterodimer structure consisting of two E proteins and two M proteins was constructed (Fig. S1). The Mutant structures (D389G, D389H, and D389S) were constructed using Pymol [16] based on WT structure. AMBER22 [17] was used to perform 500 ns of accelerated molecular dynamics (aMD) simulations of WT and all mutant structures [18]. The aa protonation states were settled using Propka3 [19] and the hydrogen bond network around the residue. The starting structures were placed in a cubic box of TIP3P water [20], the distance between the protein and box boundaries was set to a minimum of 12 Å, and counter-ions (Cl^−^) were added in order to maintain the electroneutrality of the systems. The force field employed to describe the aa residues was the FF19SB [21]. Firstly, the five-step minimization protocol was applied. The H atom, solvent water molecule, aa residue side chain, aa residue main chain, and the whole system were optimized by the steepest-descent (SD) and conjugate gradients (CG), respectively. Then, the backbone atoms of the aa residues were restrained using a harmonic constant of 5 kcal/mol/Å2, and the system was performed to heat to 300 K in NVT ensemble, and the density equilibration of 6ns was performed in NPT ensemble. Afterward, 10 ns of non-restrictive classical molecular dynamic simulations (cMD) were performed, and the average dihedral and total potential energies were computed during the last 8 ns as a reference for the aMD simulations (Table S2 and Fig. S2). Finally, 500 ns of aMD simulations were performed employing Langevin dynamics as a thermostat (collision frequency of 2 ps).

In the process of molecular dynamics simulation, the SHAKE algorithm [22] was employed to constraints all bonds involving hydrogen atoms, and the Particle Mesh Ewald (PME) method [23] was employed to calculate the electrostatic interactions of long distances with a cutoff radius of 10 Å and the time step is 2 fs.

### Animal ethics declaration

Animal care and experimental procedures were followed according to the Regulations of Experimental Animals of Sichuan Authority and approved by the Animal Ethics Committee of North Sichuan Medical College (approval number: 20,220,823).

### Statistical analysis

Measured values are expressed as means with standard deviations (SD). For viral plaque diameters analysis and the tests of a comparison of viral loads in the brain, peripheral replication kinetics, and blood clearance between the mutants and parental virus, an one-way ANOVA was used to determine statistically significant differences. Growth curves and the heparin binding assays were analyzed by two-way ANOVA analysis of variance with a multiple comparisons. All analyses were performed using SPSS software version 17.0 (SPSS, Inc., Chicago, IL, USA).

## Results

### Construction and identification of the mutants

**Fig. 1:**
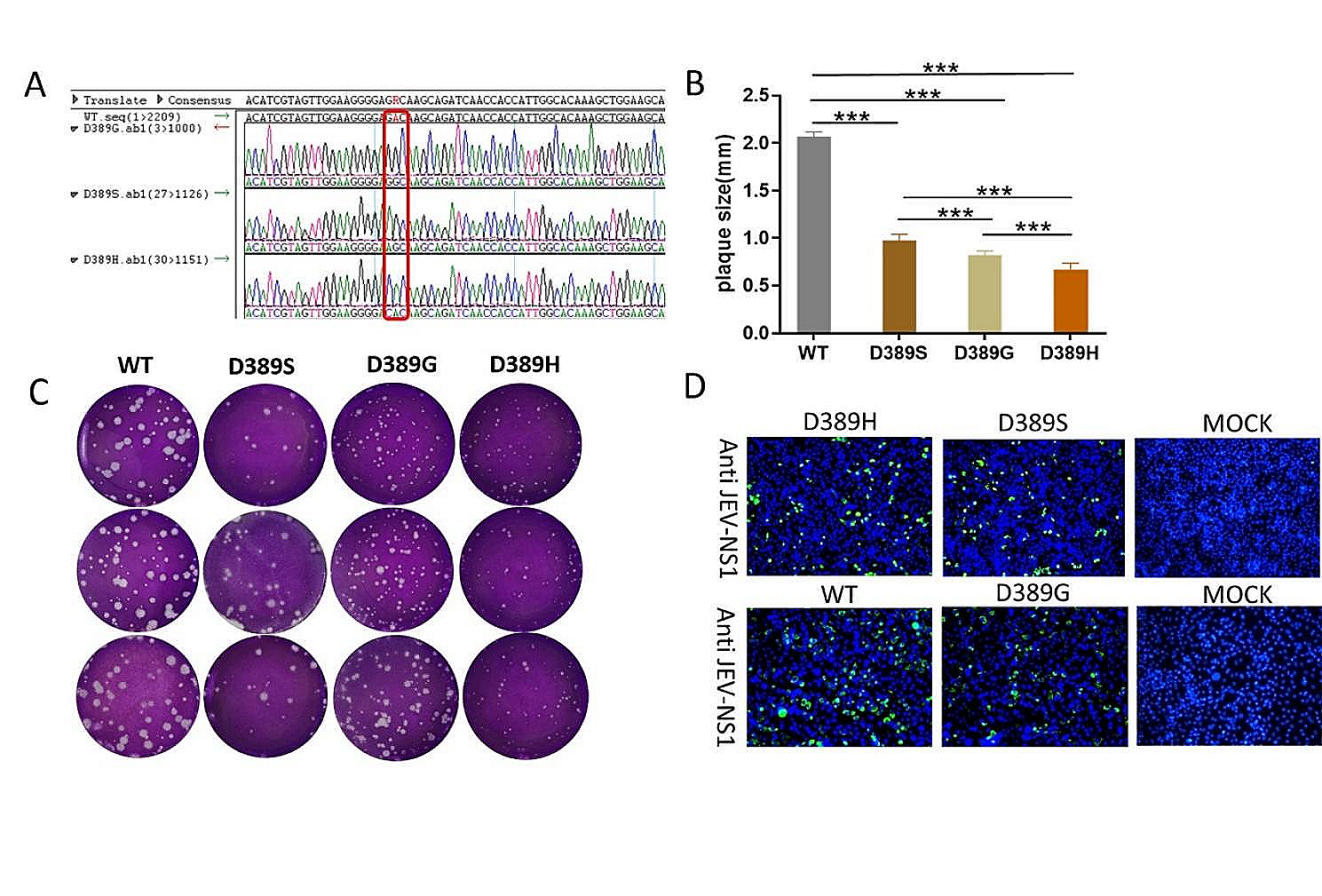
Identifying the mutants. (**A**) The sequencing results of 10 passage cultures of the virus showed that the aa of the three mutants at E389 were different from those of the WT. (**B**) Statistical analysis of viral plaque diameters of the WT virus and mutants. Three independent experiments were conducted, with 10 plaques per virus were selected at random for measuring in each experiment. The data are presented as means and SD and were tested for significance using a one-way ANOVA with multiple comparison tests (**, *P*<0.01;***, *P*<0.001; ****, *P*<0.0001). (**C**) Plaque form and size of WT, along with three mutants, D389S, D389G, and D389G, in BHK-21 cells. (**D**) The BHK-21 cells were inoculated with viruses for 48 h, four mutants were identified using the JEV NS1 protein antibodies and stained with FITC-labeled antibodies. The identifiable cells exhibited green fluorescence.DAPI was used to stain the nucleus. The data are representative of at least three independent experiments

All plasmids containing specific mutations were verified by sequencing, and the viruses used for testing were amplified by ten passages in the BHK-21 cells. The E protein-coding region of each virus was sequenced an additional time, with the results confirming that the sequences of all mutants were correct without additional mutations (Fig. 1A).

The plaque assay was used to test all viruses and compare the plaque size of different viruses in BHK-21 cells. Among the viruses, Asp at E389 is represented as WT, and those replaced by Ser, Gly and His are represented as D389S, D389G and D389H, respectively. The results indicate that the plaque diameters of the mutants D389S, D389G, and D389H were 0.97 ± 0.07, 0.82 ± 0.04 and 0.67 ± 0.06 (Fig. 1B), all of which were substantially smaller than WT (*P* < 0.0001). Among them, the plaque size of WT was the biggest, followed by those of D389S and D389G, respectively, and the plaque size of D389H was the smallest (Fig. 1C). Indirect immunofluorescence assays showed that the four kinds of mutants can be recognized by the antibodies against NS1 protein of JEV (Fig. 1D).

### Characterization of virus growth

**Fig. 2:**
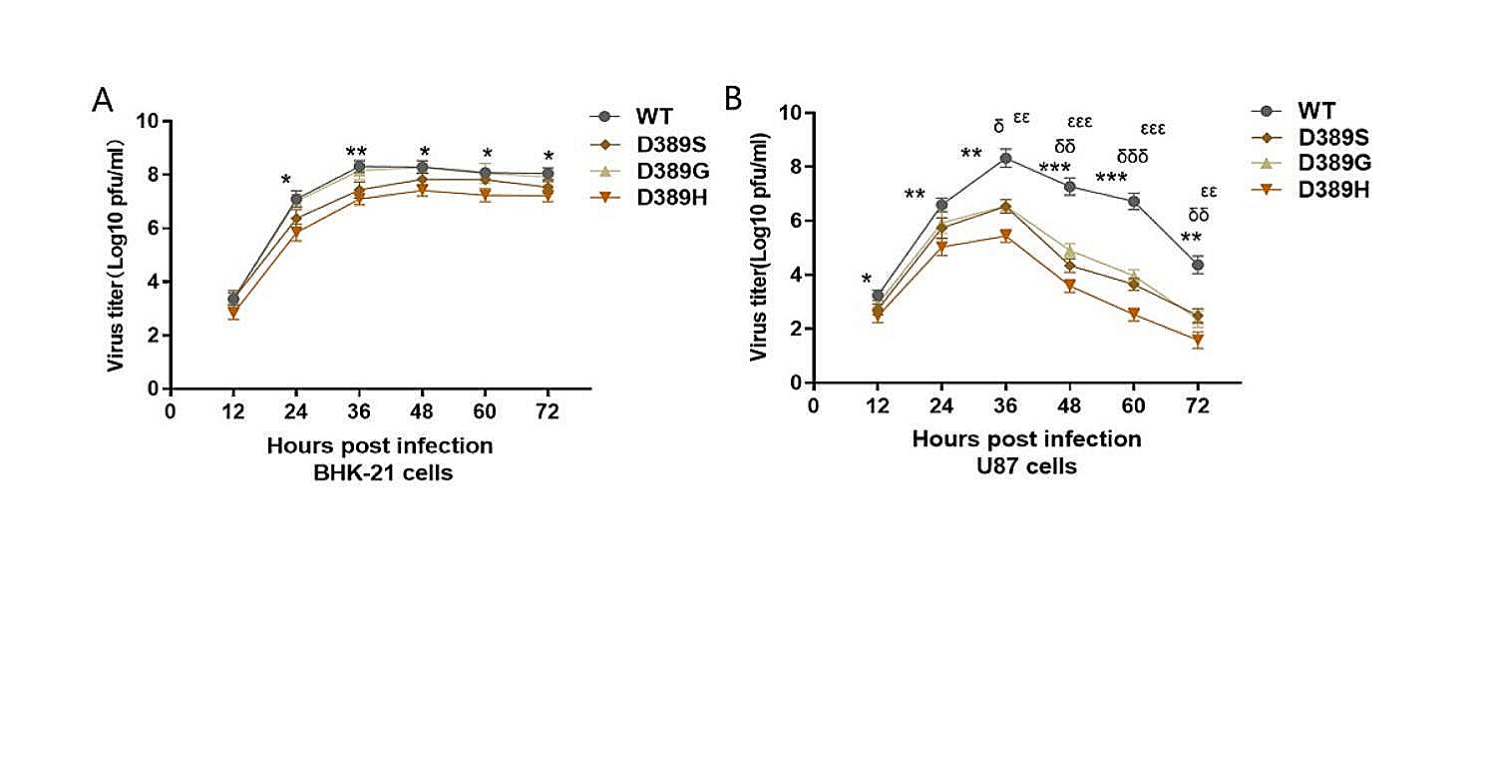
Growth curves of the mutants and the WT virus. The growth curve of the mutants was assayed in BHK-21 (**A**) and U87 cells (**B**). Viruses were infected in cell monolayers at a MOI of 0.01. Culture supernatants were collected from infected cells at 12 h intervals and were used for the estimation of viral titers in cells. The data are given as means SD from three individual experiments and were tested for significance using a two-way ANOVA with multiple comparison tests. * represents a significant difference between WT and D389H (*, *P*<0.05;**, *P*<0.01; ***, *P*<0.001), ε, between WT and D389S (ε, *P*<0.05; εε, *P*<0.01; εεε, *P*<0.001), δ, between WT and D389G (δ, *P*<0.05; δδ, *P*<0.01; δδδ, *P*<0.001)

The effects of different aa changes at the E389 site on JEV replication were measured by infecting BHK-21 cells, followed by a determination of the production of mutants and WT viruses at different time intervals after infection. The results of the growth curve showed that all four viruses exhibited similar replication capacities, reaching the peak at 48 h, although the virus titers of D389H were significantly lower than that of WT at almost all time points (*P* < 0.05), indicating that its replication capacity may be reduced (Fig. 2A). However, in U87 cells, which were more sensitive to JEV, the titer of all viruses reached its peak at 36 h, but the peak titers of the mutants were much lower than that of WT (*P* < 0.05), and the production of viruses decreased rapidly after reaching the peak. Suggesting that the ability of mutants to replicate in nerve cells is more severely affected (Fig. 2B). Impaired viral replication may be one of the mechanisms leading to its attenuated virulence in mice.

The acidity/alkalinity of the side chain of the E389 residues is associated with viral neuroinvasiveness.

For many aa sites that are important for JEV virulence, the aa properties of virulent and attenuated strains were compared, and it was found that the mutation of basic aa was crucial for JEV virulence. Of the three mutants we successfully rescued, Asp in E389 of the WT was replaced by Ser, Gly, and His, respectively. Asp is acidic, Ser and Gly aliphatic, while His is alkaline. To investigate whether the acidity/alkalinity of E389 is related to JEV virulence, we measured the LD_50_ (PFU) values of neurovirulence (Table 1) and neuroinvasiveness (Table 2) of all mutants. Low LD_50_ (PFU) values indicate high degrees of virulence.

In mice inoculated with the viruses by i.c. route, the LD_50_ value of WT was similar to that of the three mutants, all of which showed strong neurovirulence. However, compared to that of WT JEV SA14, the neuroinvasiveness of the D389S, D389G, and D389H decreased, and D389G and D389H were more significant. These results indicating that the D-to-G/H mutation significantly weakened WT. It suggested that the properties of residues 389 side chain plays an important role in JEV neuroinvasiveness.

**Table 1:** Neurovirulence of the mutants in 3-week-old mice inoculated by the i.c. route

Virus dilution multiple	Neurovirulence assay in 3-week-old mice (death/total)					
		WT	D389S	D389G	D389H	
10^2^		6/6	6/6	NA	6/6	
10^3^		6/6	6/6	6/6	6/6	
10^4^		6/6	6/6	6/6	6/6	
10^5^		6/6	4/6	5/6	5/6	
10^6^		2/6	3/6	3/6	2/6	
10^7^		NA	NA	0/6	NA	
Initial titer of virus (PFU/ml)		1.5 × 10^7^	6.5 × 10^6^	1.4 × 10^7^	1.5 × 10^7^	
LD_50_(PFU)		0.8	0.4	0.6	1.03	

NA: not available, represent the experiments were not carried out

**Table 2:** Neuroinvasiveness testing of the mutants by i.p. inoculation of 3-week-old mice

Virus dilution multiple	Neuroinvasiveness assay in 3-week-old mice (death/total)			
	WT	D389S	D389G	D389H
10^0^	NA	6/6	5/6	2/6
10^1^	6/6	6/6	2/6	1/6
10^2^	5/6	1/6	0/6	0/6
10^3^	1/6	0/6	0/6	0/6
10^4^	2/6	0/6	0/6	0/6
10^5^	0/6	NA	NA	NA
Initial titer of virus (PFU/ml)	1.18 × 10^6^	1.3 × 10^7^	8.9 × 10^6^	9 × 10^6^
LD_50_(PFU)	1.18 × 10^3^	1.63 × 10^5^	1.03 × 10^6^	>4.5 × 10^6^

NA: not available, represent the experiments were not carried out

### Spread of mutant JEVs in 3-week-old Kunming mice

**Fig. 3:**
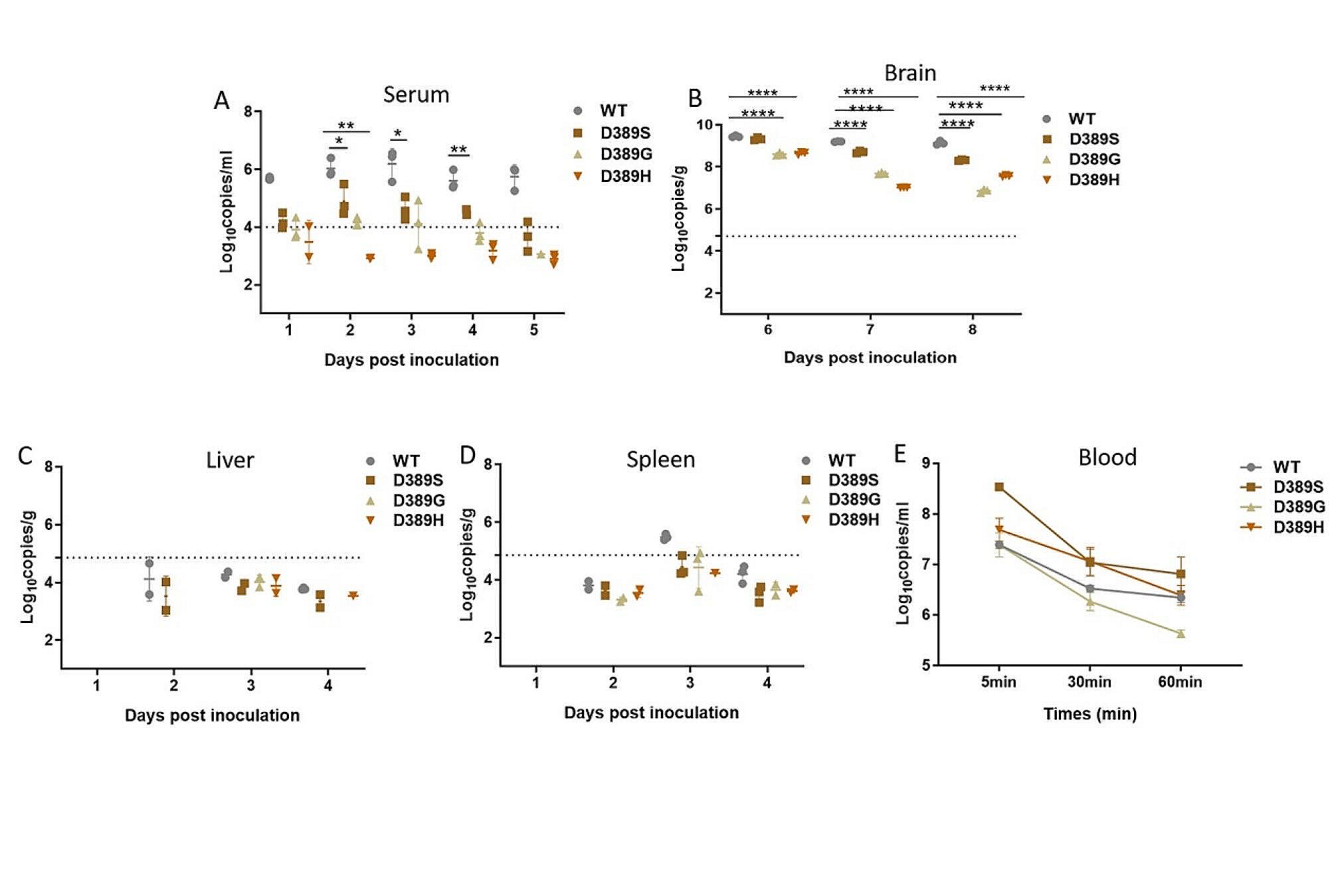
A comparison of viral loads in the brain, peripheral replication kinetics, and blood clearance between the mutants and parental virus. 3-week-old Kunming mice were inoculated by the i.p route with three mutants and WT (${10}^{6 }$PFU) respectively and divided into four groups (*n* = 24). Serum (**A**), brain (**B**), liver (**C**), and spleen (**D**) were collected at specific time spots, and the gene copies of the samples were analyzed by qRT-PCR. Each sample was a fusion of three mouse tissues. The minimum limit of the test was ${10}^{4.7}$ copies/ml for brain, ${10}^{4}$copies/ml for serum and ${10}^{5 }$copies/g for liver and spleen, both represented by dashed lines. (E) The mutants and parental virus (${10}^{6}$ PFU) by i.v route were inoculated to 6-week-old Kunming mice, and blood was collected at 5, 30, and 60 min and assayed for viral loads by qRT-PCR. Each data represents the mean of three samples. The data are representative of at least three independent experiments, and error bars indicate the SD. Significance was calculated using a one-way ANOVA with multiple comparison tests. * represents a significant difference between WT and mutants (*, *P*<0.05;**, *P*<0.01; ***, *P*<0.001; ****, *P*<0.0001)

The mechanism of the loss of neuroinvasiveness of the viruses was investigated by examining the kinetics of viruses spread from the peripheral tissue of inoculation compared with that of the WT. The 3-week-old Kunming mice were inoculated with 10^6^ PFU mutants and WT by i.p. route. The brains of the mice were collected at 6, 7, and 8 dpi, and the livers and spleens were collected at 2, 3, and 4 dpi. To detect viremia, blood was collected from 1 to 5 dpi. The mice inoculated with WT produced detectable viremia (Fig. 3A) at 1 to 5 d after inoculation, resulting in the spread of the virus across the blood-brain barrier to the brain, and high viral titers were observed 6 to 8 dpi (Fig. 3B). At d 3, low viral replication (3 × 10^5^ copies/g) was also detected in the spleen (Fig. 3D). However, virus was not detected in the liver at any point in time (Fig. 3C). Mice inoculated with the mutant D389S and D389G both detected low levels of viriemia from 1 to 3 dpi, but after this time, the virus copies decreased rapidly and was undetectable at d 5. However, mice inoculated with the attenuated mutant D389H had weak viremia detected only on d 1 (Fig. 3A), and no infectious virus was found in the liver or spleen of all mice inoculated with the mutants at any time (Fig. 3C and D). In the brain, viral replication was detected in all three mutants and showed a decrease after d 6. It should be noted that gene copies in the brains of mice inoculated with D389H showed an increase in d 8, which may be caused by individual differences in mice or the reason why mice inoculated with the mutant D389H showed clinical symptoms later than other viruses. Overall, the viral loads in brain tissue of all mutants were lower than that of the WT at d 8, with D389G and D389H being more significant (Fig. 3B).

### Blood clearance kinetics of mutant JEVs

The degree of viremia is mainly determined by two factors: on the one hand, the viral load that enters the blood circulation through peripheral replication, and on the other hand, the efficiency of virus clearance from the blood by the reticuloendothelial system. The rapid removal of viruses from the blood may be related to increased affinity for GAGs during viral adsorption [24, 25]. The mice were inoculated with 10^6^PFU virus by the i.v route, and blood samples were collected at 5, 30, and 60 min p.i. The results showed about a 11-fold reduction of viremia in the WT-inoculated animals within 60 min, compared with about a 52, 56, and 19-fold reduction in the mutant D389S, D389G, and D389H, respectively, during the same period. However, D389S decreased rapidly from 5 min to 30 min after virus entry into the blood, while the trend gradually stabilized from 30 min to 60 min (Fig. 3E).

### Effect of heparin on virus binding on BHK-21 cells

**Fig. 4:**
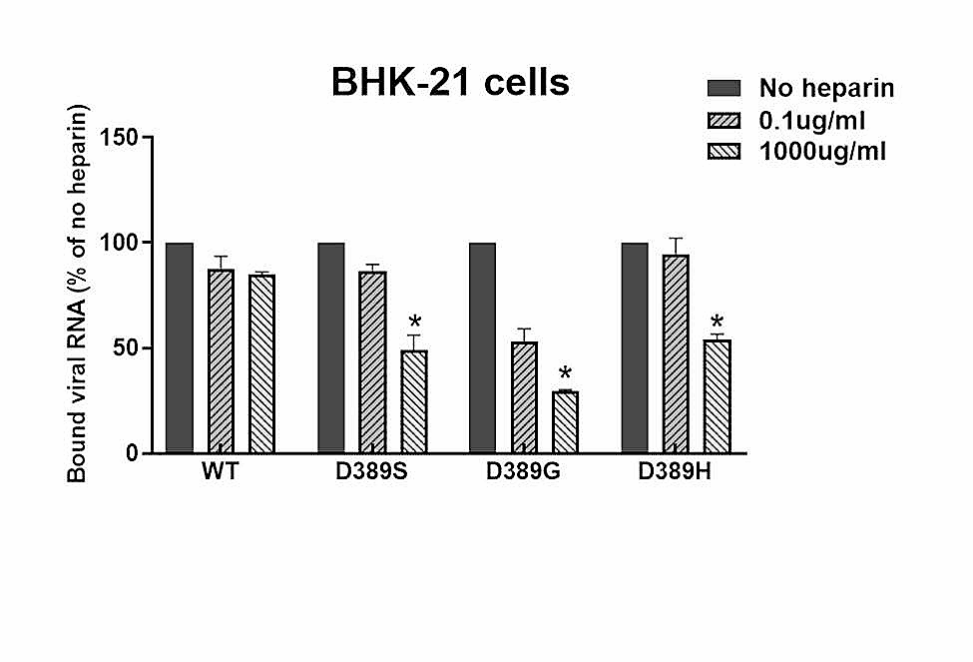
Impact of heparin on the binding of JEV mutants to BHK-21 cells

It has been reported that heparin is a highly sulfated proteoglycan whose molecular structure is similar to that of heparin sulfate, which is widely present in cells and extracellular matrix, and GAGs have an obvious inhibitory effect on positively charged attenuated mutants [26, 27].To assay the effect of GAGs on the E389 mutants binding to BHK-21 cells, a dose-dependent heparin binding inhibition assay was performed for D389S, D389G, and D389H on BHK-21 cells. As shown in Fig. 4, high-dose heparin significantly decreased the binding of attenuated mutants to BHK-21 cells, and this effect was most significant for D389G. However, the highest dose of heparin had little inhibitory effect on the adsorption of WT to BHK-21 cells. These results suggested that the mutant D389G was more susceptible to heparin inhibition.

The virus was precoated with different concentrations of heparin at 37 °C for 30 min and then cooled at 4 °C for 5 min, and the mixture was inoculated into a monolayer of pre-cooled BHK-21 cells and cultured at 4 °C for 1 h. RNA copy numbers of the bound viruses were assayed by qRT-PCR, and GAPDH was used as a control. The percentage of copies of each virus treated with heparin relative to those not treated with heparin is shown above. The data are presented as means and SD from three independent experiments (*n* = 3) and were tested for significance using a one-way ANOVA with multiple comparison tests. *, *P*<0.05.

### The flexibility of the E protein affects the neuroinvasiveness of mutant JEVs

**Fig. 5:**
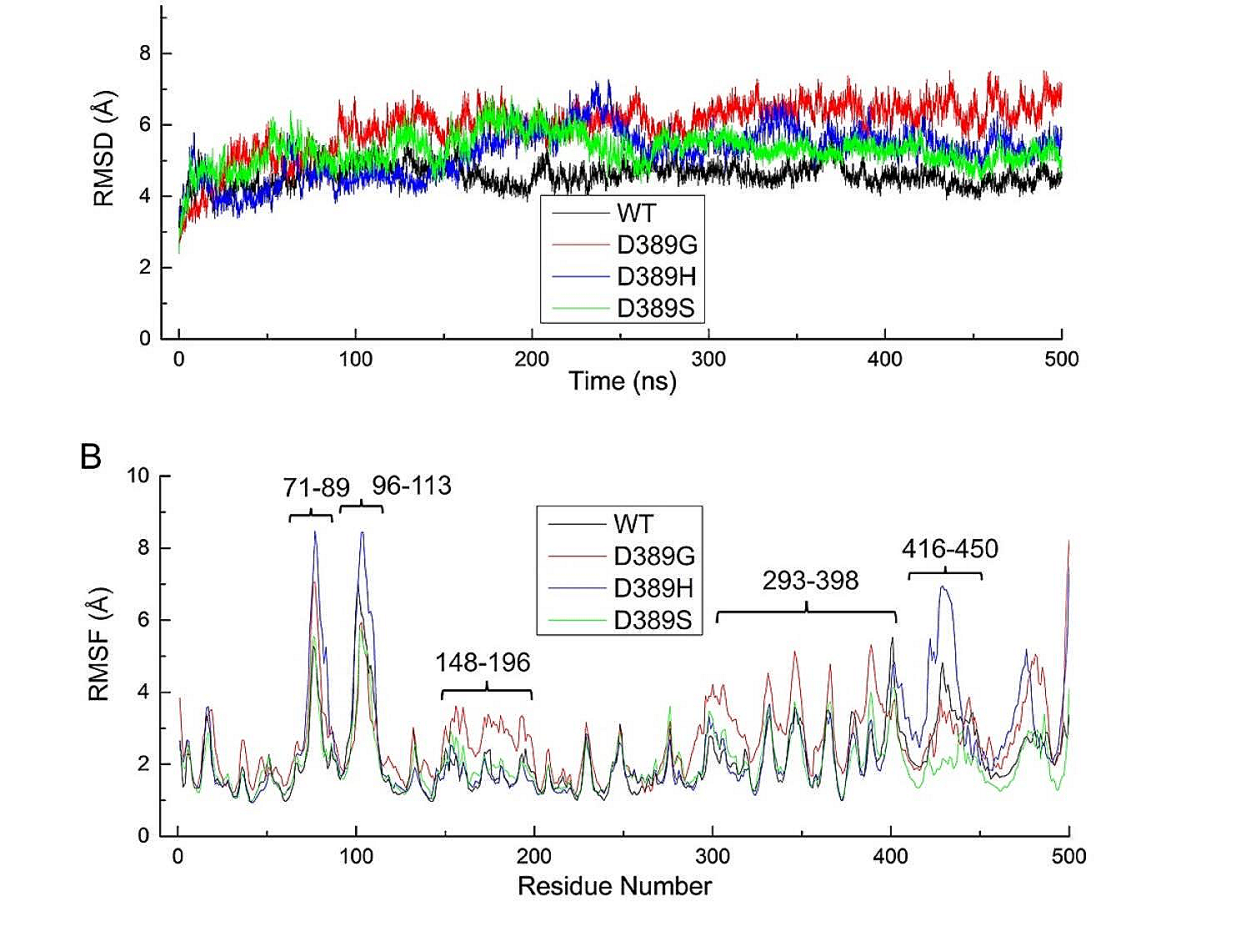
RMSD (**A**) and RMSF (**B**)

To explore the effect of aa E389 mutation on E protein conformation dynamics, 500 ns of accelerated molecular dynamics (aMD) simulations for WT JEV SA14 and its mutants (D389G, D389H, and D389S) were performed. The root mean square deviation (RMSD) analysis of backbone atoms relative to the initial model was used to detect the conformational changes of the E protein and the root mean square fluctuation (RMSF) analysis was employed to compare the fluctuation of each residue of the E protein in different mutants. The RMSD analysis showed that compared with the parental strain JEV SA14, RMSD of each mutant was increased, indicating more conformational changes and enhanced flexibility in the mutants (Fig. 5A). RMSF analysis (Fig. 5B) allowed us to identify that the increased flexibility of D389H was mainly associated with two regions of the structural domain II (DII) and one region of the domain III (DIII). In the DII, one of the regions involves the residues 71–89. The maximum RMSF values were about 8.5 Å (D389H) and 5.3 Å (WT), respectively. The other region includes residues 96–113, whose maximum RMSF values were 8.4 Å (D389H) and 7.0 Å (WT), respectively. The RMSF maximums of 416–450 residues in domain III were 7.0 Å (D389H) and 4.8 Å (WT), respectively. However, the increased flexibility of D389G was mainly related to domains I (DI) and III (DIII), where the RMSF values of residues 148–196 and 293–398 were higher than those of the WT, D389H, and D389S.

In (A), changes in conformations are illustrated for different times (0, 100, 200, 300, 400, and 500 ns), showing that the WT system presents lower RMSD values, suggesting limited changes in conformation. In (B), the residues with more fluctuations are displayed (71–89, 96–113, 148–196, 293–398, and 416–450). Different color numbers are represented by the residues involved in DI (green), DII (red), DIII, and helical stem (blue).

**Fig. 6:**
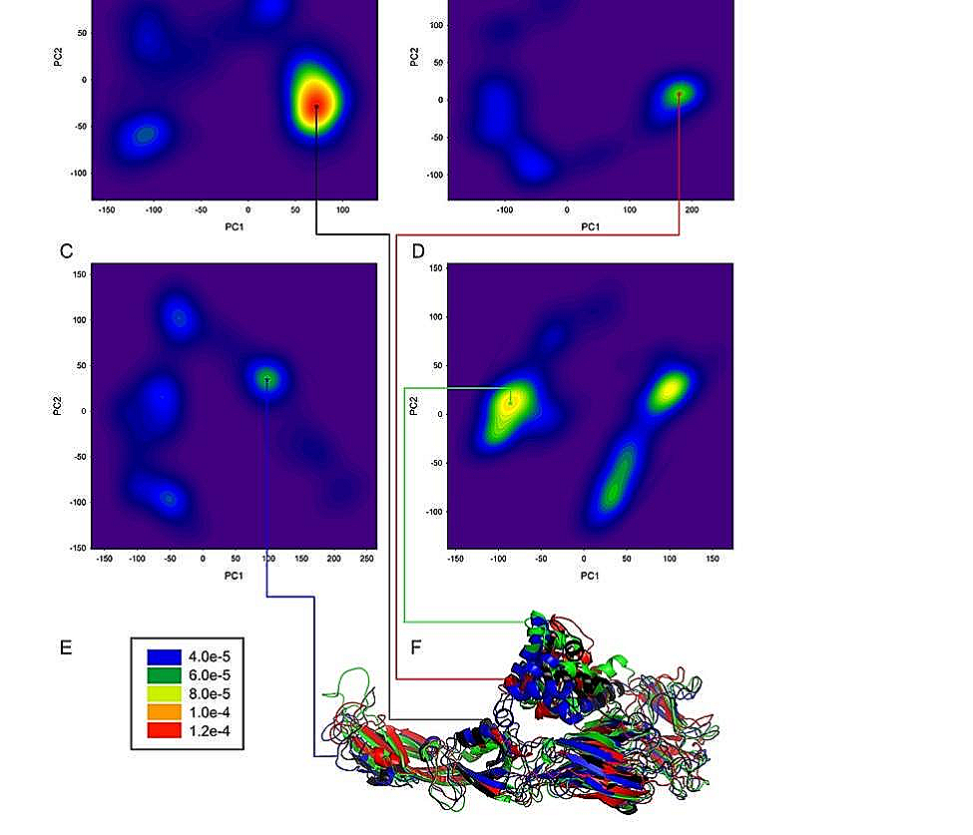
Histogram of PCA for WT (**A**), D389G (**B**), D389H (**C**), and D389S (**D**), and the corresponding color scale bar (**E**). The alignment of the minimal ensemble conformations of the systems WT (black), D389G (red), D389H (blue) and D389S (green), for clarity, only one E protein for each system is given (F)

Principal components analysis showed that the mutants changed conformational minimum states (Fig. 6, Fig S3-S6), and the bending movement of E protein was enhanced, indicating that the flexibility of both mutants D389G and D389H were enhanced, and D389G was more significant, which was consistent with the results of RMSF analysis.

## Discussion

Attenuation of viral virulence is achieved through serial virus passage in the sensitive cells or animal models, a process that may cause adaptive mutations of individual aa of the virus, resulting in attenuation of viral virulence. This view is supported by flaviviruses [24, 28]. Adaptive mutants of MVE were selected by successive passages in SW13 cells and were characterized by the following features: expression of small plaques in SW13 [3] cell monolayers, increased sensitivity to heparin, decreased neuroinvasiveness in mice [24]. In this study, we serially passaged the WT strain JEV SA14 in BHK-21 cells and accidentally discovered the E389 (D to G) mutant, which was significantly reduced in neuroinvasiveness compared with the WT JEV. However, the site was still aspartic acid in the attenuated strain SA14-14-2.

The E protein of flavivirus is crucial to the virulence of the virus. We previously demonstrated that chimeric viruses obtained by replacing the E protein of JEV SA14 with that of attenuated strain SA14-14-2 exhibit attenuated neurovirulence and neuroinvasiveness [13]. Flavivirus molecules responsible for impacting virulence and receptor binding have been attributed by structural prediction to two clusters: one with E residues 138 and 49 on the surface of structural domain I and the other with E residues 306 and 390, located in the apical position of the DE and FG loops in structural domain III [29]. Continuous passaging of the JEV, shows that two mutations, E138K and D389G, repeatedly occur in several mutants and, resulting in its small plaque and decreased virulence [30]. Our data support the finding that a single mutation of E389 could substantially attenuate the neuroinvasiveness of the WT strain JEV SA14. By analyzing the structure of the E protein, E389 was found to be located in the receptor binding region of DIII. We used full-length cDNA cloning technology to mutate E389 to aa with different charges through site-specific mutagenesis and successfully saved all the mutants. The growth characteristics and neuroinvasiveness of the mutants were significantly different from those of the WT strain JEV SA14. After the Asp of E389 is replaced by aliphatic aa (Gly, Ser) or weakly basic aa (His), the neuroinvasiveness of the virus will be affected in different degrees. We speculate that the neuroinvasiveness of virus may also be related to the acidity/alkalinity of this site. However, only three aa with different chemical properties were selected as representatives in this study. It is necessary to introduce more mutations to elucidate the relationship between the acidity/alkalinity of E389 aa and the JEV neuroinvasiveness.

It has been shown that many pathogenic microorganisms utilize GAG as an attachment vector that enhances the attachment of viral particles to the surface of target cells prior to engagement with the primary receptor, impacting their tissue tropism [31–33]. The most common GAG is heparan sulfate, a highly sulfated form of heparin sulfate that inhibits GAG-mediated viral adsorption [34, 35]. In vitro heparin binding inhibition experiment, it was found that a high dose of heparin inhibited the three mutants more significantly than the WT strain. This result is most likely due to the proximity of the E389 site to the GAG binding domain, which affects the interaction between viruses and GAGs, and the positive charge mutations make JEV surface proteins more easily bind to negatively charged GAGs on the cell surface, thus showing a stronger affinity for heparin.

According to E protein structure analysis, we found that replacing Asp at the E389 site would disrupt the stability of E protein, resulting in a certain degree of damage to viral replication in cultured cells, and this result was also confirmed in yellow fever virus (YFV) and its live-attenuated form-YFV 17D [36]. By comparing the differences in growth kinetics between mutants and WT, we found that that compared with WT strain JEV SA14, the replications of mutant D389H was weakened in BHK-21 cells, but in U87 cells, the replications of all three mutants were inhibited to different degrees. This suggested that the replication capacities of the mutants in nerve cells were significantly decreased, which was consistent with the decrease of neuroinvasiveness. The virus showed different growth kinetics phenotypes in mammalian cells and nerve cells, which may be due to the participation of E protein in the nerve cell taxis of the virus, making the difference in growth kinetics between mutants and WT more obviously in U87 cells. The structural simulation results of the E protein showed that the E protein of the alkaline mutants was more flexible than that of the WT. The alterations in flexibility induced by D389H mutation were mainly concentrated on the fusion ring of domain II and the helical stem of the E protein (E-H1, E-H2, and E-H3), which regions were mainly related to the stability of the virus. Increased flexibility may lead to a lower population of H bonds, making the virus less structurally stable, but the reason why poor stability leads to decreased neuroinvasiveness remains to be further studied. The flexibility alterations of the E protein induced by D389G mutation were mainly located in DI, the linker of DI and DIII, and the DIII region, which may act as points of attachment to cellular integrins and be associated with binding GAGs.However, in a normal conformational structure, most of the residues implicated in these regions are buried, and conformational rearrangements would be required to expose aa for binding GAGs [14]. The conformational structures of mutant D389G in these regions were more flexible, which increased the aa associativity to GAGs, resulting in a decrease in the amount of virus entering the brain, which is likely to be responsible for the attenuated neuroinvasiveness phenotype.

The rapid clearance of the GAG-sensitive variant of JEV from the bloodstream prevented the sufficient magnitude and duration of viremia required for viral entry into the brain, which may account for the mechanism of attenuation of the neuroinvasiveness of the GAG-binding variant of JEV. The mutants were cleared at a faster rate compared to the WT strain. Among them, D389G and D389S decreased more significantly. However, D389G showed a significant decline within 60 min after the virus entered the blood, while D389S showed a significant decline in the first 30 min, and gradually flattened out between 30 and 60 min. These results suggested that the blood clearance process may allow the mutants acquiring lower viremia and the resulting viral load of the virus into the brain tissue was reduced. However, different mutants have different clearance rates in the blood, which may be due to their different sensitivity to immune factors in the blood.

The mechanism of how flaviviruses breach the blood-brain barrier to enter the brain parenchyma is very complex. The main mechanism is that virus invasion leads to infection of brain capillary endothelial cells, which enter the brain parenchyma through diffusion or endocytosis [37, 38]. Whether the virus can generate high titer virus and persistent viremia in the body is the key to the strength of its neuroinvasive ability. Our results showed that the mutants have a lower viremia than WT and a shorter duration. The mice inoculated with WT developed detectable viremia within 1–5 d after infection, while mice inoculated with the mutants D389S and D389G showed low levels of viremia on 1–3 d, after which the virus content decreased rapidly, whereas the mice inoculated with D389H only detected at very low levels of viremia on d 1. However, the mutants were not detected in the liver and spleen at either time point after peripheral inoculation, and the mice inoculated with WT was only detected at very low levels in the spleen on d 3, indicating that impaired replication in peripheral tissue was not the primary reason of mutants attenuated. It is interesting that the mice inoculated attenuated mutant D389H were only detected at very low levels of viremia on d 1, and 107copies/g of the virus were detected in the brain of the mice on d 8. A possible explanation for this phenomenon is a transient virulence atavism of D389H in the mouse brain or a viral induction of a transient viral replication enhancement event mediated by a regulatory inflammatory pathway in the host. On the other hand, in normal animals, the production of type I IFN plays an important role in the resistance of mice to flavivirus infection, so viremia in extra neurologic tissues and viral replication in peripheral organs are rarely detected in mice with normal immune function at 3-week-old [39, 40].

Gene knockout mice with defective IFN-α receptor function (IFN-α-R^−/−^ mice) do not respond to IFN-α/β at all and are highly susceptible to infection with flavivirus, resulting in high titer viremia and obvious clinical symptoms [41, 42]. Further testing of the differences in peripheral tissue replication kinetics between the mutants and WT strain in IFN-α-R^−/−^ mice is meaningful.

## Conclusion

In this study, mutation at the E389 site (Asp→His) significantly reduced the neuroinvasiveness of WT strain JEV (SA14), comparable to the effects of previously reported mutations at the E107 and E138 sites. Our data demonstrated that increased flexibility of the E protein of aa at E389 decreased JEV neuroinvasiveness, and that the mechanism of viral attenuation may also be related to the increase of GAG affinity. However, it is interesting that the site of the attenuated strain SA14-14-2 is conserved, and whether its virulence will be further reduced if the E389 site of SA14-14-2 is mutated remains to be studied.

In summary, this study demonstrated that E389 is an important virulence-related site, which is worthy of further study and has important significance for exploring the mechanism of virulence attenuation.

### Electronic supplementary material

Below is the link to the electronic supplementary material.

Supplementary Material 1

## Data Availability

No datasets were generated or analysed during the current study.
