# Supplementary material for: The mutation of Japanese encephalitis virus envelope protein residue 389 attenuates viral neuroinvasiveness
Source: Virol J. 2024 Jun 5;21:128. doi: 10.1186/s12985-024-02398-8 (PMC11151615; doi:10.1186/s12985-024-02398-8)
Supplement: Supplementary file 1 — Supplementary Material 1 [file 12985_2024_2398_MOESM1_ESM.docx]

Supplementary Information

Table S1. Primers used for PCR and qRT-PCR.

| Designation of primers | Sequence of primers | Usage of primers |
| --- | --- | --- |
| F1 | CAGGCGCCATGAAGTTGTCGAATTTCC | Underlined is the *KasI* enzymatic site used for the insertion of the fragment |
| F389G | TTGGAAGGGGAG**G**CAAGCAGATCAACCACCATTG | Bolded is the mutated base（A→G） |
| R389G | GTTGATCTGCTTG**C**CTCCCCTTCCAACTACG | Bolded is the mutated base（T→C） |
| F389S | TTGGAAGGGGA**AG**CAAGCAGATCAACCACC | Bolded are the mutated bases（GA→AG） |
| R389S | GTTGATCTGCTTG**CT**TCCCCTTCCAACTACG | Bolded are the mutated bases（TC→CT） |
| F389H | TTGGAAGGGGA**C**ACAAGCAGATCAACCACC | Bolded is the mutated base（G→C） |
| R389H | GTTGATCTGCTTGT**G**TCCCCTTCCAACTACG | Bolded is the mutated base（C→G） |
| R1 | CAGATCTGACTCCGCACACGCCTTCC | Underlined is the *BglII* enzymatic site used for the insertion of the fragment |
| JEV NS5-F | GCTCAACGAGACCACCAACT | qRT-PCR |
| JEV NS5-R | TCAGCGAACACTGCTCCAAG | qRT-PCR |


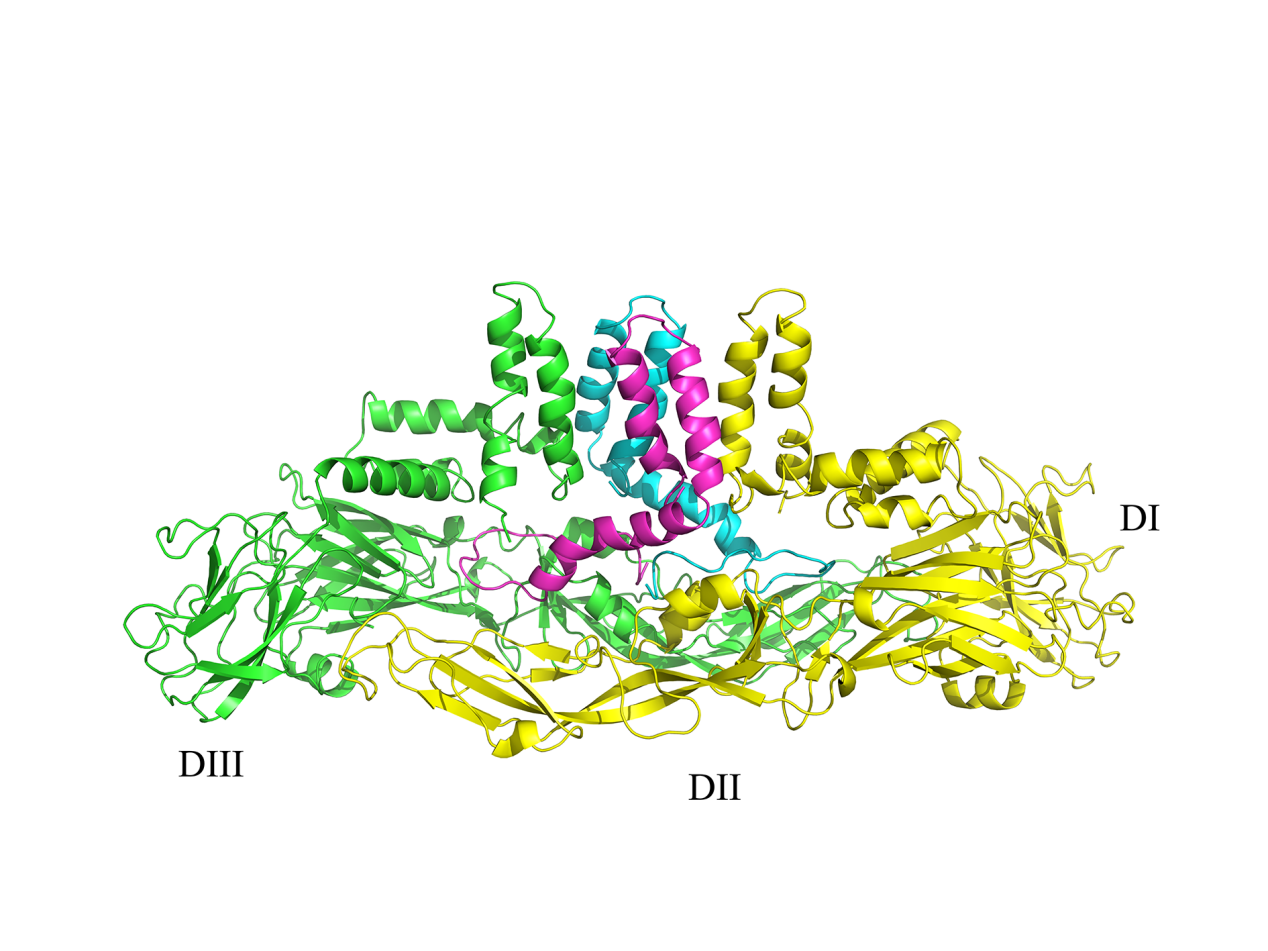


Fig. S1. WT heterodimer structure containing two E proteins and two M proteins. The E protein is represented by green and yellow, and the M protein is represented by blue and magenta.

Table S2. Variables and parameters used in the aMD simulation for all systems.

| Systems | | WT | D389G | D389H | D389S |
| --- | --- | --- | --- | --- | --- |
| Variables | Residues | 1148 | 1148 | 1148 | 1148 |
|  | Total Atoms | 183338 | 183330 | 183329 | 183332 |
|  | Waters | 55368 | 55368 | 55361 | 55366 |
|  | Ions | 4Cl^−^ | 6Cl^−^ | 6Cl^−^ | 6Cl^−^ |
|  | EPtot (kcal/mol) | −556767.6 | −556757.3 | −556709.5 | −556819.3 |
|  | DIHED (kcal/mol) | 5875.4 | 5847.2 | 5873.6 | 5871.9 |
| Calculated Parameters | Ethreshd | 9893.4 | 9865.2 | 9891.6 | 9889.9 |
|  | Alphad | 803.6 | 803.6 | 803.6 | 803.6 |
|  | Ethreshp | −527433.5 | −527424.5 | −527376.9 | −527486.2 |
|  | Alphap | 29334.1 | 29332.8 | 29332.6 | 29333.1 |


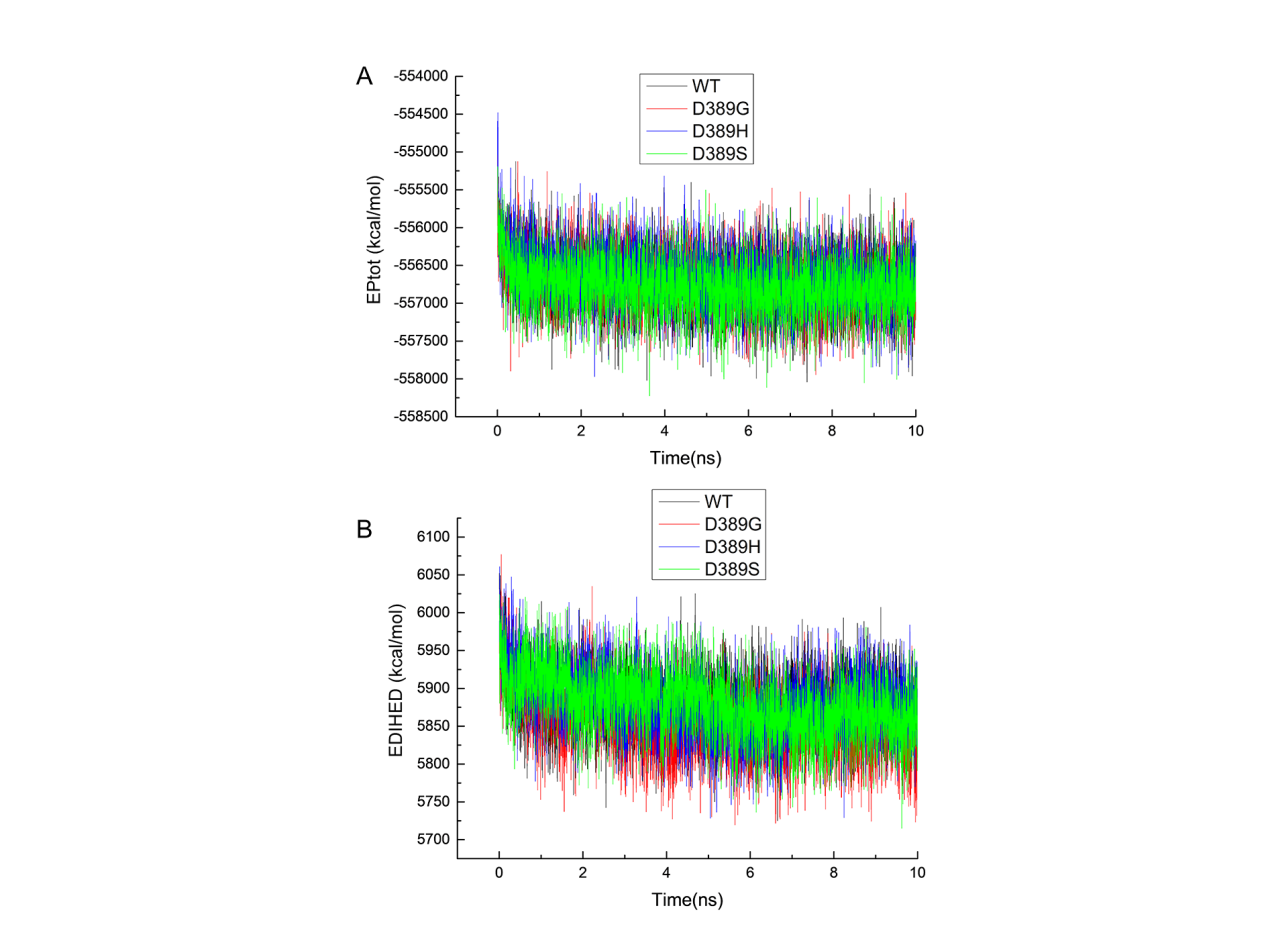


Fig. S2. Eptot (A) and DIHED (B) during 10ns of cMD for the systems.


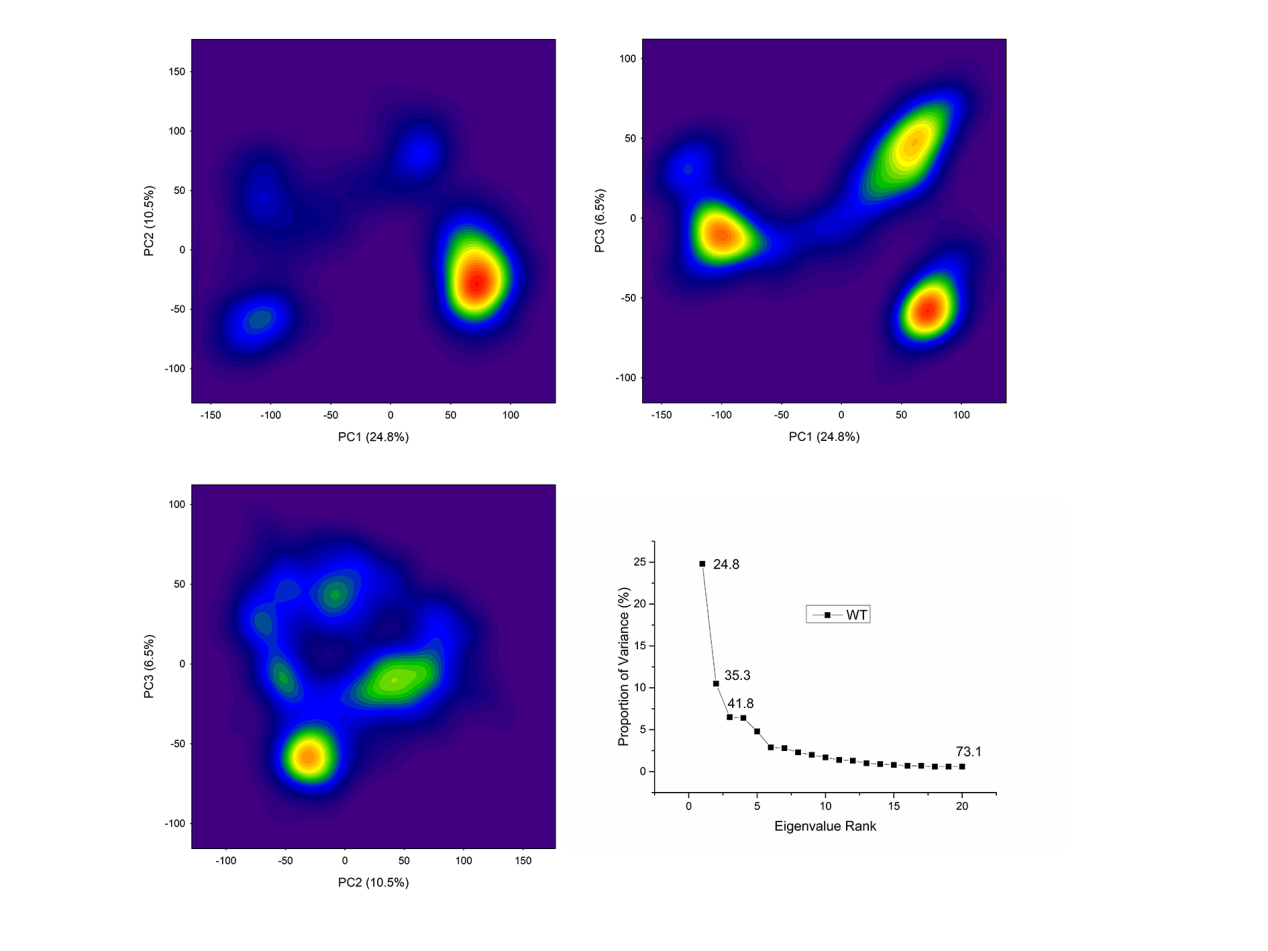


Fig. S3. PCA data of WT


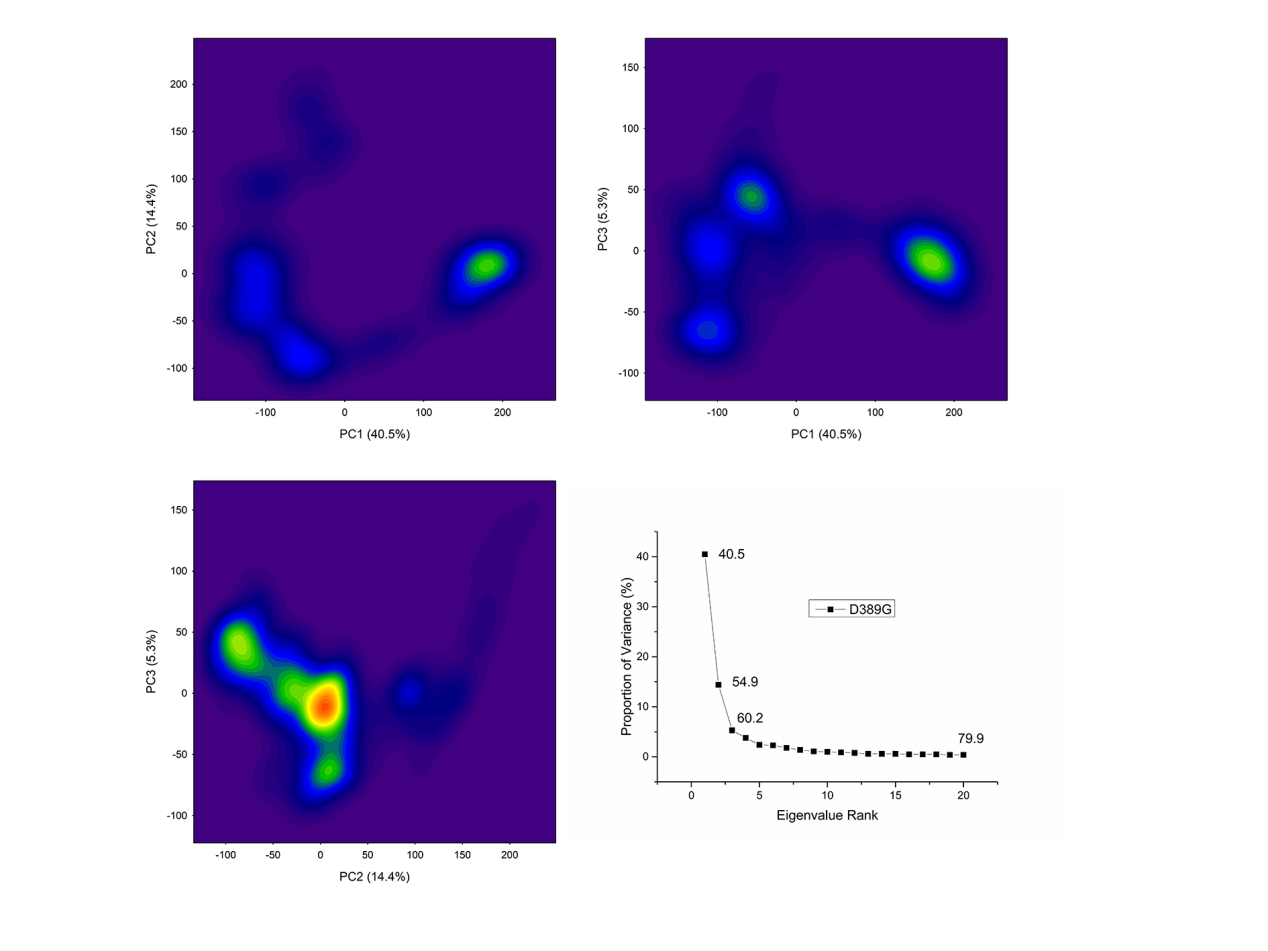


Fig. S4. PCA data of D389G


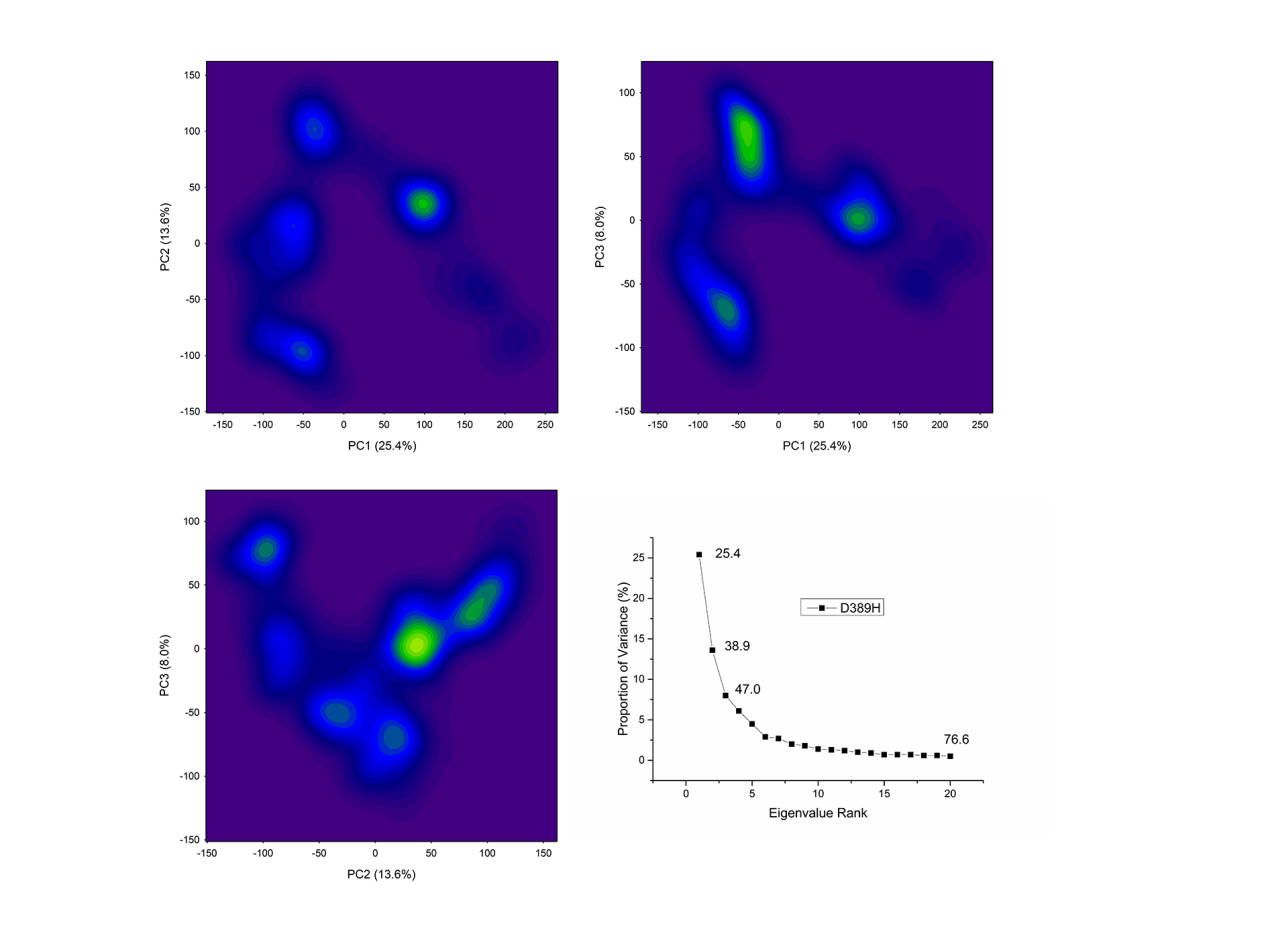


Fig. S5. PCA data of D389H


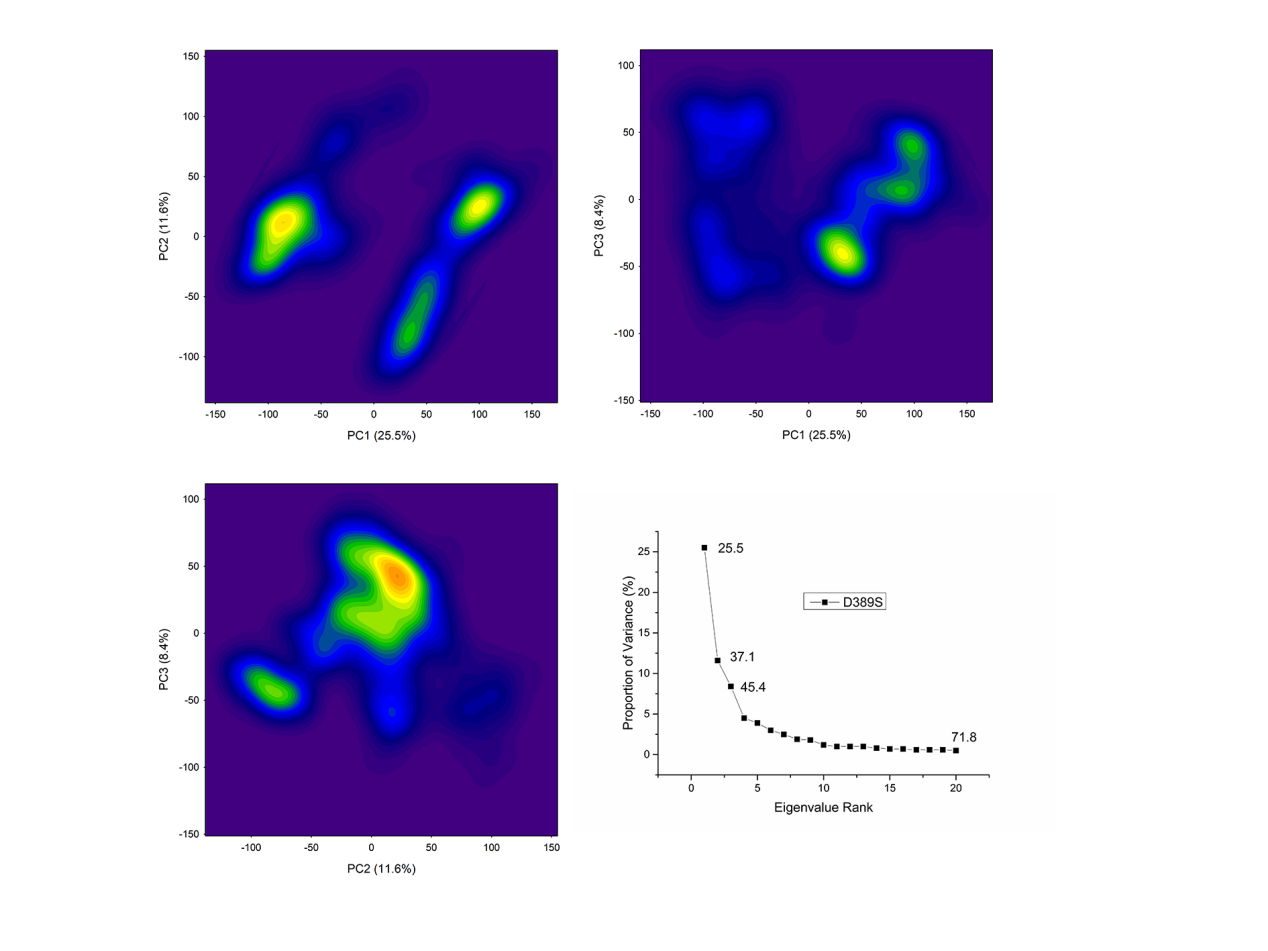


Fig. S6. PCA data of D389S
